# Prevalence, Risk Factors and Outcomes of Velamentous and Marginal Cord Insertions: A Population-Based Study of 634,741 Pregnancies

**DOI:** 10.1371/journal.pone.0070380

**Published:** 2013-07-30

**Authors:** Cathrine Ebbing, Torvid Kiserud, Synnøve Lian Johnsen, Susanne Albrechtsen, Svein Rasmussen

**Affiliations:** 1 Department of Obstetrics and Gynaecology, Haukeland University Hospital, Bergen, Norway; 2 Clinical Foetal Physiology Research Group, Department of Clinical Science, University of Bergen, Norway; Vanderbilt University, United States of America

## Abstract

**Objectives:**

To determine the prevalence of, and risk factors for anomalous insertions of the umbilical cord, and the risk for adverse outcomes of these pregnancies.

**Design:**

Population-based registry study.

**Setting:**

Medical Birth Registry of Norway 1999–2009.

**Population:**

All births (gestational age >16 weeks to <45 weeks) in Norway (623,478 singletons and 11,263 pairs of twins).

**Methods:**

Descriptive statistics and odds ratios (ORs) for risk factors and adverse outcomes based on logistic regressions adjusted for confounders.

**Main outcome measures:**

Velamentous or marginal cord insertion. Abruption of the placenta, placenta praevia, pre-eclampsia, preterm birth, operative delivery, low Apgar score, transferral to neonatal intensive care unit (NICU), malformations, birthweight, and perinatal death.

**Results:**

The prevalence of abnormal cord insertion was 7.8% (1.5% velamentous, 6.3% marginal) in singleton pregnancies and 16.9% (6% velamentous, 10.9% marginal) in twins. The two conditions shared risk factors; twin gestation and pregnancies conceived with the aid of assisted reproductive technology were the most important, while bleeding in pregnancy, advanced maternal age, maternal chronic disease, female foetus and previous pregnancy with anomalous cord insertion were other risk factors. Velamentous and marginal insertion was associated with an increased risk of adverse outcomes such as placenta praevia (OR = 3.7, (95% CI = 3.1–4.6)), and placental abruption (OR = 2.6, (95% CI = 2.1–3.2)). The risk of pre-eclampsia, preterm birth and delivery by acute caesarean was doubled, as was the risk of low Apgar score, transferral to NICU, low birthweight and malformations. For velamentous insertion the risk of perinatal death at term was tripled, OR = 3.3 (95% CI = 2.5–4.3).

**Conclusion:**

The prevalence of velamentous and marginal insertions of the umbilical cord was 7.8% in singletons and 16.9% in twin gestations, with marginal insertion being more common than velamentous. The conditions were associated with common risk factors and an increased risk of adverse perinatal outcomes; these risks were greater for velamentous than for marginal insertion.

## Introduction

Velamentous cord insertion is diagnosed when the umbilical vessels insert into the membranes before they reach the placental margin. This results in the umbilical vessels lacking the protection of Wharton’s jelly for the section between the insertion and the placental margin. A marginal cord insertion is where this distance is reduced to a minimum, but the insertion site is supported by very little placental tissue. Velamentous insertion of the umbilical cord has been associated with an increased risk of adverse perinatal outcomes [1]–[3]. Velamentous vessels are associated with vasa praevia (where the vessels traverse the internal os of the cervix in front of the leading foetal part), a condition that is associated with high perinatal mortality when it is not diagnosed prenatally [4]. Therefore, ultrasound screening for vasa praevia in high-risk populations (e.g. twin pregnancies and pregnancies conceived with the aid of assisted reproductive technology) has been suggested as a cost-effective measure [5], but a better understanding of velamentous and marginal cord insertions is needed [6].

Abnormal cord insertion seems to be associated with impaired development and function of the placenta, and thus influences foetal growth [7], [8] and has been linked to placenta praevia and pregnancy-induced hypertension [3]. The altered development of the placenta with anomalous cord insertion may influence the relationship between birthweight and placental weight, but this has yet to be confirmed. Neither is it known whether there is an increased risk of recurrence of anomalous cord insertion in a subsequent pregnancy.

The existing data on risk factors and perinatal outcome in pregnancies with anomalous cord insertion are conflicting [2], [9]. Velamentous and marginal insertions are reported to occur in 0.5–2.4% and 8.5% of all pregnancies, respectively [2], [3], [10], with the prevalence being higher in multiple pregnancies [11] and in pregnancies conceived with the aid of assisted reproductive technology [12]. However, these prevalence rates are derived from hospital registers, which might be influenced by selection bias, and population-based studies are lacking.

The aims of the present study were thus 1: to establish a population-based prevalence of velamentous and marginal insertions of the umbilical cord, 2: to identify risk factors for anomalous cord insertion, and 3: assess the risk for adverse perinatal outcomes associated with these conditions.

## Materials and Methods

### Ethics Statement

The Regional Committee for Medical and Health Research Ethics West approved the study protocol (approval no. REC West 2011/949), and waived the need for written informed consent form the participants, since the data were analysed anonymously.

A population-based registry study was performed of all singleton births at gestational weeks 16–45 in Norway during the period 1999–2009 using data from the Medical Birth Registry of Norway. Twin pregnancies were also studied. Registration of birth is compulsory in Norway, and the registry contains information on all births since 1967 based on information taken from a form completed by the attending midwife or physician shortly after delivery. Information regarding whether the umbilical cord insertion into the placenta was normal, marginal, velamentous, or had vessel anomalies was requested on the form used since 1999. The attending midwife weighs the placenta with the membranes and umbilical cord attached. The registry also holds information regarding the maternal health before and during pregnancy, paternal age, delivery, placental weight, birthweight and perinatal outcomes. Voluntary notification of all pregnancies conceived with the aid of assisted reproductive technology has been included in the registry since 1988; the inclusion of this information became compulsory since 2001. The gestational age (in weeks) was based on ultrasound dating when available (96.8%), and otherwise based on the mother’s last menstrual period. Smoking habits were registered after obtaining informed consent. The information of smoking habits at the start of pregnancy was collected at the first prenatal visit. Perinatal mortality was defined as death before birth or within 7 days after birth. Parity was defined as the number of previous deliveries, and a birth before gestational week 37 was defined as preterm. Serious malformations are in the MBRN defined by specific Q diagnoses in the ICD10 system (see List S1). Notification of any malformation was done by the attending physician at birth or at the neonatal care unit. The association between anomalous cord insertion and birthweight discordance between twins (defined as the absolute difference in birthweight between the twins expressed as a percentage of the weight of the larger twin) was determined, with a difference of >20% regarded as discordant [11].

### Statistical Analyses

The odds ratios (ORs) and 95% confidence intervals (95% CIs) for velamentous and marginal cord insertions were estimated for different risk factors using logistic regression analyses. The same method was applied to assess the OR for adverse outcomes in pregnancies with anomalous cord insertion. Only parous women were included when calculating the OR for anomalous cord insertion after caesarean section. In analyses including two or more births from the same woman, multilevel regression analysis was used in order to avoid biased risk estimates and standard errors caused by the hierarchical structure of the data (births within women). In the multivariate analyses possible confounders such as maternal age, parity, chronic disorders and smoking were added to the models as indicator variables. When assessing the risk for adverse pregnancy outcomes in pregnancies with anomalous cord insertion, the analyses were stratified for gestational age. In order to calculate the OR for a repeat velamentous or marginal cord insertion in the subsequent pregnancy, the first and second births of each woman were linked using national identification numbers.

The birthweight discordance was analysed in 11,263 pairs of twins where the birthweight of both twins was known. Birthweight percentiles were calculated for gestational age (≥20 weeks of gestation), gender and parity. Gestational age (≥20 weeks) and gender-specific percentiles for placental weight and birthweight/placenta-weight ratio were based on published reference ranges [13]. Statistical analyses were carried out using the Statistical Package for Social Sciences for Windows (version 20.0, SPSS, Chicago IL, USA) and the MlWin programme (Centre for Multilevel Modelling, University of Bristol, UK).

## Results

The prevalence rates of velamentous and marginal cord insertions in a total of 623,478 singleton pregnancies were 1.5% (*n* = 9,500; 95% CI = 1.5–1.6%) and 6.3% (*n* = 39 403; 95% CI = 6.3–6.4%), respectively. In the population of singletons 0.4% were born at gestational age week 17–21 (n = 2797), 0.3% at 22–26 (n = 1985), 0.7% at 27–31 (n = 4240), 4.6% at 32–36, 93% at 37–42 (n = 583555), and 0.3% at >42 weeks (n = 2110). Among the pairs of twins (*n* = 11,263), the prevalence rates of velamentous and marginal cord insertions were 5.9% and 10.9%, respectively, while the ORs in twin births were 4.0 (95% CI = 3.7–4.3) and 1.8 (95% CI = 1.7–1.9), respectively. Maternal and pregnancy descriptive statistics of the twin pregnancies are shown in Table S1.

Tables 1 and 2 list the maternal and pregnancy characteristics as risk factors for anomalous cord insertion in singletons. In total, 10,208 singleton pregnancies were conceived with the aid of assisted reproductive technology, and these had an OR of 2.5 (95% CI = 2.3–2.8) for velamentous insertions and 1.6 (95% CI = 1.5–1.7) for marginal insertions. After adjustment for maternal age and parity these values were slightly modified, at OR = 2.2 (95% CI = 1.9–2.4) and OR = 1.4 (95% CI = 1.3–1.5), respectively. Adding maternal chronic conditions (asthma, hypertension and diabetes) and smoking as indicator variables did not influence the estimate, and were therefore not included in the final model. We found no increased risk of anomalous cord insertion in twins conceived with the aid of assisted reproduction technology compared with naturally conceived twins (OR = 0.9, 95% CI = 0.8–1.1).

**Table 1:** Odds ratios (ORs) for velamentous and marginal cord insertions according to maternal and pregnancy characteristics, based on 623,478 singleton pregnancies in Norway recorded between 1999 and 2009 and adjusted for maternal age and parity.

Maternal and pregnancy characteristics			Velamentous cord insertion						Marginal insertion					
		Total (n)		Yes (n)	%	OR	95% CI		Yes (n)	%	OR	95% CI		
**Maternal age (years)** ¥	<20	14932		222	1.49				1025	6.86				
	20–24	92848		1318	1.42	1.06	0.92	1.23	5685	6.12	0.91		0.85	0.98
	25–29	204640		2890	1.41	1.06	0.92	1.21	12448	6.08	0.94		0.88	1.00
	30–34	206515		3162	1.53	1.22	1.06	1.40	13080	6.33	1.01		0.95	1.08
	35–39	89292		1601	1.79	1.50	1.30	1.74	6070	6.80	1.12		1.04	1.20
	40+	15205		306	2.01	1.75	1.46	2.09	1095	7.20	1.22		1.11	1.33
**Parity** *	0	256136		4292	1.68				17190	6.71				
	1	221788		3228	1.46	0.81	0.78	0.85	13488	6.08	0.88		0.85	0.90
	2	102295		1415	1.38	0.73	0.68	0.77	6253	6.11	0.85		0.83	0.88
	3	29453		394	1.34	0.67	0.60	0.74	1734	5.89	0.80		0.76	0.84
	4+	13806		171	1.24	0.59	0.50	0.69	738	5.35	0.70		0.65	0.76
**Assisted reproductive technology**	No	613270		9127	1.49				38442	6.27				
	Yes	10208		373	3.65	2.16	1.94	2.41	961	9.41	1.43		1.34	1.53
**Fetal gender** * ¥	Male	319257		4453	1.39				19978	6.26				
	Female	302353		5045	1.67	1.20	1.15	1.25	19421	6.42	1.03		1.01	1.05
**Smoking**	No	412424		6367	1.54				27555	6.68				
	Yes	102883		1827	1.78	1.19	1.12	1.25	6732	6.54	0.99		0.96	1.01
	NA	108171		1306	1.21	0.77	0.73	0.82	5116	4.73	0.69		0.67	0.71
**Previous caesarean**	No	316219		4406	1.39				18844	5.96				
	Yes	51123		802	1.57	1.10	1.02	1.19	3369	6.59	1.10		1.06	1.14
**Vaginal bleeding in pregnancy**	No	595813		8707	1.46				37349	6.27				
	Yes	27665		793	2.87	1.98	1.84	2.23	2054	7.42	1.20		1.14	1.25

CI, confidence interval; NA, not answered;

¥ not adjusted for maternal age;

* not adjusted for parity.

**Table 2:** ORs for velamentous and marginal cord insertions according to maternal conditions and folate and multivitamin supplementation, based on 623,478 singleton pregnancies in Norway and adjusted for maternal age and parity.

Maternal condition			Velamentous cord insertion					Marginal cord insertion				
		Total (n)	Yes (n)	%	OR	95% CI		Yes (n)	%	OR	95% CI	
**Rheumatoid arthritis**	No	621715	9473	1.52				39305				
	Yes	1763	27	1.53	0.99	0.68	1.45	98	5.56	0.86	0.70	1.06
**Asthma**	No	597177	9018	1.51				37655				
	Yes	26301	482	1.83	1.23	1.12	1.34	1748	6.65	1.06	1.01	1.11
**Chronic hypertension**	No	620012	9430	1.52				39058				
	Yes	3466	70	2.02	1.27	1.00	1.61	345	9.95	1.61	1.44	1.80
**Diabetes**	No	612850	9273	1.51				38609	6.30			
	Type 1	2812	62	2.20	1.45	1.12	1.87	200	7.11	1.13	0.98	1.31
	Type 2	1267	24	1.89	1.22	0.82	1.83	102	8.05	1.29	1.06	1.58
	GDM	5996	129	2.15	1.40	1.17	1.66	439	7.32	1.16	1.06	1.28
	Unspecified	553	12	2.17	1.38	0.78	2.45	53	9.58	1.54	1.16	2.05
**Epilepsy**	No	618708	9418	1.52				39097				
	Yes	4770	82	1.72	1.13	0.99	1.30	306	6.42	1.02	0.91	1.14
**Folate supplementation**	No	522113	7866	1.51				33359				
	Yes	101365	1634	1.61	1.02	0.97	1.08	6044	5.96	0.91	0.88	0.93
**Multivitamin supplementation**	No	547777	8278	1.51				34849				
	Yes	75701	1222	1.61	1.03	0.97	1.09	4554	6.02	0.92	0.89	0.95

OR, odds ratio; CI, confidence interval; GDM, gestational diabetes mellitus.

Paternal age was not associated with the occurrence of anomalous cord insertion (data not shown). The first childbirth exhibited an increased risk of anomalous cord insertion. Sex was a significant determinant, with female foetuses being linked to an increased risk of velamentous cord insertion (OR = 1.2, 95% CI = 1.2–1.3) and–to a lesser extent–marginal insertion (OR = 1.03, 95% CI = 1.0–1.05).

Smoking at the start of pregnancy slightly increased the risk of velamentous cord insertion (OR = 1.2, 95% CI = 1.1–1.3) but not marginal insertion (Table 1). Women who did not respond to the question about smoking (17.3%, *n* = 108,171) had a reduced OR for anomalous cord insertion (OR = 0.8, 95% CI = 0.7–0.8). Previous experience of caesarean section delivery was associated with a slightly increased risk of anomalous cord insertion (Table 1).

Vaginal bleeding in pregnancy was associated with doubled risk of anomalous cord insertion (Table 1). The risk of anomalous cord insertion did not differ significantly when assessed for gestational age at the time of bleeding (i.e. bleeding during the first, second or third trimester of pregnancy; <13 weeks: OR = 2.1, 95% CI = 1.9–2.3; 13–28 weeks: OR = 2.0, 95% CI = 1.8–2.3; >28 weeks: OR = 1.7, 95% CI = 1.4–2.0 for velamentous cord insertion).

Our examination of whether maternal medical conditions affects the risk of anomalous cord insertion (Table 2) revealed that maternal asthma, chronic hypertension, and type 1 and gestational diabetes were associated with increased risk of velamentous cord insertion (apart from marginal insertion, which did not show an increased risk of type 1 diabetes), even after controlling for maternal age, parity, folate and multivitamin supplementation and smoking. The analyses indicated that folate and multivitamin supplementation did not affect the umbilical cord insertion site (velamentous OR = 1.02, 95% CI = 1.0–1.1; Table 3). This was not influenced by including maternal medical conditions, age, parity and smoking in the model.

**Table 3:** ORs for velamentous and marginal cord insertions in pregnancies subsequent to pregnancies with a velamentous or marginal insertion (singleton pregnancies only), adjusted for maternal age and between 1999 and 2009.

Index pregnancy		Next pregnancy									
Cord insertion		Velamentous cord insertion					Marginal cord insertion				
	n	n	%	OR	95% CI		n	%	OR	95% CI	
Normal	163250	2102	1.3	1			8756	5.4	1		
Velamentous	2958	68	2.3	1.8	1.4	2.3	215	7.3	1.3	1.2	1.5
Marginal	12814	205	1.6	1.2	1.1	1.4	919	7.2	1.4	1.3	1.5

OR, odds ratio adjusted for maternal age; CI, confidence interval.

The ORs were increased for velamentous and marginal cord insertions in a subsequent pregnancy (Table 3). The risk estimates were negligibly influenced by maternal age and interpregnancy interval. To further elucidate the association between anomalous cord insertion and other placental conditions, we assessed the occurrence of placental abruption and placenta praevia following a pregnancy with velamentous cord insertion. No increased risk of these conditions was found in the subsequent pregnancies (data not shown).

The OR of placental abruption was two- to threefold higher in pregnancies with anomalous cord insertion (Table 4). This was not changed by adding maternal chronic medical conditions, assisted reproductive technology or smoking as indicator variables, thus these factors were not included in the final model. The risk for placenta previa was three-to fourfold higher in pregnancies with velamentous cord insertion. Whether the pregnancy was conceived by assisted reproductive technology modified this estimates slightly: OR 3.56 (95% CI = 2.9–4.3), while including maternal chronic hypertension or diabetes to the model had no influence on the risk estimates.

**Table 4:** ORs for pregnancy complications according to umbilical cord insertion site, based on 623,478 singleton pregnancies and adjusted for maternal age and parity.

Cord insertion site		Condition	Yes (n)	Total (n)	%	OR	95% CI	
**Velamentous**	no	**Abruptio placenta**	2443	613978	0.40			
	yes		98	9500	1.03	2.60	2.12	3.18
**Marginal**	no		2311	584075	0.40			
	yes		230	39403	0.58	1.48	1.29	1.69
**Velamentous**	no	**Placenta previa**	1713	613978	0.28			
	yes		102	9500	1.07	3.71	3.03	4.55
**Marginal**	no		1614	584075	0.28			
	yes		201	39403	0.51	1.82	1.57	2.11
**Velamentous**	**no**	**Pre-eclampsia**	22426	613978	3.65			
	yes		536	9500	5.64	1.51	1.39	1.65
**Marginal**	no		20901	584075	3.58			
	yes		2061	39403	5.23	1.45	1.38	1.52
**Velamentous**	no	**Preterm birth**	577266	613978	5.98			
	yes		8399	9500	11.59	2.03	1.90	2.16
**Marginal**	no		549240	584075	5.96			
	yes		36425	39403	7.56	1.28	1.23	1.33

OR, odds ratio; CI, confidence interval.

The risk of pre-eclampsia was increased with both velamentous and marginal cord insertions, and the increased risk persisted when stratified for parity = 0 and parity≥1 (OR = 1.5 and 1.6, respectively). When stratifying for gestational age, the risk of pre-eclampsia was increased in term (OR = 1.4, 95% CI = 1.2–1.5) but not preterm pregnancies (OR = 1.14, 95% CI = 0.96–1.35) with velamentous cord insertion. Adjusting for maternal smoking at the start of pregnancy, or chronic medical conditions did not significantly change the estimates and thus these factors were not included in the final model. The increased risk for pre-eclampsia remained in both term- and preterm-births for pregnancies with marginal insertion (OR 1.3, 95% CI = 1.3–1.4, and OR 1.6, 95% CI = 1.5–1.8).

In total, 37,813 births (6.1%) were preterm, and 20,786 (3.3%) were spontaneous preterm births. Of all preterm births 7.3% were in the lowest gestational age group (week 17–21), 5.2% in week 22–26, 11.2% week 27–31, and 76.1% in the moderate preterm group (week 32–36). The risk of spontaneous and non-spontaneous preterm births was doubled in pregnancies with velamentous cord insertion (Table 4), with spontaneous preterm birth having an OR of 1.9 (95% CI = 1.7–2.0).

The data presented in Table 5 indicate that anomalous cord insertion was associated with an increased risk of acute caesarean section but not of operative vaginal delivery. The association of increased risk of acute caesarean section with velamentous insertion slightly differed when stratified for 4 categories of gestational age; Gestational age <32 weeks OR = 2.4 (95% CI = 1.8–3.0), moderate preterm group (32–36 weeks) OR = 1.5 (95% CI = 1.3–1.8), term pregnancies (37–42 weeks) OR = 1.6 (95% CI = 1.5–1.7) and gestational age >42 weeks OR = 2.1 (95% CI = 1.0–4.7). Adjustment for maternal age, parity, diabetes, chronic hypertension, asthma or smoking did not change the risk estimates. The risk of elective caesarean section was borderline significantly increased in the preterm group (<32 weeks OR = 1.6, 95% CI = 1.0–2.6), whereas in the late preterm and term groups the risk of elective caesarean was not increased (OR = 1.1, 95% CI = 0.9–1.3 and OR 1.1, 95% CI = 1.0–1.2).

**Table 5:** ORs for adverse outcome (delivery) according to umbilical cord insertion site, based on 623,478 singleton pregnancies and adjusted for maternal age and parity.

Cord insertion site		Condition	Yes (n)	Total (n)	%	OR	95% CI	
**Velamentous**	no	**Caesarean acute**	48830	613978	7.95			
	yes		1346	9500	14.17	1.80	1.69	1.91
**Marginal**	no		45916	584075	7.86			
	yes		4260	39403	10.81	1.37	1.32	1.42
**Velamentous**	no	**Caesarean elective**	34119	613978	5.56			
	yes		600	9500	6.32	1.11	1.02	1.22
**Marginal**	no		32121	584075	5.50			
	yes		2598	39403	6.59	1.19	1.14	1.25
**Velamentous**	no	**Forceps delivery**	7895	613978	1.29			
	yes		140	9500	1.47	1.05	0.89	1.25
**Marginal**	no		7460	584075	1.28			
	yes		575	39403	1.46	1.09	1.00	1.19
**Velamentous**	no	**Vacuum delivery**	43657	613978	7.11			
	yes		652	9500	6.86	0.87	0.76	0.99
**Marginal**	no		41577	584075	7.12			
	yes		2732	39403	6.93	0.92	0.88	0.96
**Velamentous**	no	**Operative vaginal delivery**	50778	613978	8.27			
	yes		783	9500	8.24	0.90	0.83	0.97
**Marginal**	no		48316	584075	8.27			
	yes		3245	39403	8.24	0.94	0.90	0.98
**Velamentous preterm**	no	**Breech presentation**	4205	36712	11.45			
	yes		199	1101	18.07	1.69	1.45	1.988
**Velamentous term**	no		18049	577266	3.13			
	yes		492	8399	5.86	1.84	1.67	2.02
**Velamentous preterm**	no	**Transverse lie**	533	36712	1.45			
	yes		29	1101	2.63	1.82	1.23	2.67
**Velamentous term**	no		1354	577266	0.23			
	yes		39	8399	0.46	1.93	1.39	2.69

OR, odds ratios; CI, confidence interval.

We also found an increased risk of breech and transverse presentations for both conditions, which did not change when stratified for term or preterm birth, or when adjusted for placenta praevia (Table 5).

Velamentous cord insertion was associated with an increased risk of a 5-minute Apgar score of <7 and transferral to a neonatal intensive care unit (Table 6). When stratified for gestational age at birth (<32, 32–36, 37–42, and >42 weeks), the risk of a low Apgar score was not significantly increased in the youngest preterm group (OR = 1.2, 95% CI = 0.9–1.5), but it was increased by 40 and 80% in the moderately preterm and term group, respectively (OR = 1.4, 95% CI = 1.0–1.8 and OR = 1.8, 95% CI = 1.5–2.0, respectively).

**Table 6:** ORs for adverse perinatal outcomes according to umbilical cord insertion site, based on 623,478 singleton pregnancies and adjusted for maternal age and parity.

Cord insertion site	Condition	Yes (n)	Total (n)	%	OR	95% CI	
**Velamentous No**	**5 min Apgar score <7**	9892	613978	1.61			
Yes		290	9500	3.05	1.87	1.66	2.10
**Marginal No**		9515	584075	1.63			
Yes		667	39403	1.69	1.02	0.94	1.11
**Velamentous No**	**Transferal to NICU**	50192	613978	8.18			
Yes		1359	9500	14.31	1.83	1.72	1.94
**Marginal No**		47451	584075	8.12			
Yes		4100	39403	10.41	1.29	1.25	1.34
**Velamentous No**	**Perinatal death**	4729	613978	0.77			
Yes		156	9500	1.64	2.14	1.83	2.52
**Marginal No**		4584	584075	0.78			
Yes		301	39403	0.76	0.97	0.87	1.09
**Velamentous No**	**Serious malformations**	16822	613978	2.74			
Yes		407	9500	4.28	1.56	1.41	1.72
**Marginal No**		15996	584075	2.74			
Yes		1233	39403	3.13	1.14	1.07	1.20
**Velamentous No**	**Birth weight centile <10th**	36768	467482	7.87			
Yes		1050	7488	14.02	1.88	1.76	2.01
**Marginal No**		34886	444365	7.85			
Yes		2932	30605	9.58	1.24	1.19	1.29
**Velamentous No**	**Placenta weight centile<10th**	46711	451243	10.35			
Yes		1222	7260	16.83	1.73	1.63	1.84
**Marginal No**		44461	428626	10.37			
Yes		3472	29877	11.62	1.13	1.09	1.17
**Velamentous No**	**BW/PW ratio centile<10th**	70911	451243	15.71			
Yes		1223	7260	16.85	1.07	1.01	1.14
**Marginal No**		67303	428626	15.70			
Yes		4831	29877	16.17	1.03	1.00	1.06

NICU, neonatal intensive care unit; BW/PW, birthweight/placental weight; OR, odds ratio; CI, confidence interval.

The risk of perinatal death was doubled in pregnancies with velamentous cord insertion relative to normal cord insertion. When stratified for gestational age at birth (<32, 32–36, >37 weeks), the OR for perinatal death in the term group was 3.3 (95% CI = 2.5–4.3) for velamentous cord insertion and 1.4 (95% CI = 1.2–1.7) for marginal cord insertion, whereas no increased risk of perinatal death was found in the preterm groups (velamentous insertion OR = 0.9, 95% CI = 0.6–1.1, and 1.2 (95% CI = 0.8–1.9), for <32 and 32–36 weeks, respectively). Both adjustment for neither maternal medical conditions, nor smoking significantly influence the risk estimates and these factors were thus not included in the final model.

Velamentous and marginal insertions were associated with an increased risk of serious foetal malformations, OR = 1.6 (95% CI = 1.4–1.8) and OR = 1.2 (95% CI = 1.1–1.2), respectively. Including maternal age, parity, and smoking and chronic conditions as indicator variables in the model had no influence on the estimates.

The risk of the foetus being small for gestational age (birthweight <10^th^ percentile) was increased for both conditions, whereas the risk of placental weight <10^th^ percentile and birthweight/placenta weight <10^th^ percentile was only increased for velamentous cord insertion.

Velamentous cord insertion in one or both twins was associated with an increased risk of birthweight discordance (OR = 1.8, 95% CI = 1.6–2.1).

## Discussion

In the Norwegian birth population, the incidence of velamentous cord insertion was 1.5% (95% CI = 1.5–1.6) in singleton pregnancies, which is lower than the value of 2.4% found in a recent hospital study [3]. We found that the two conditions–velamentous and marginal cord insertions–followed the same pattern of risk factors, and also shared being risk factors of adverse outcomes. We found that velamentous insertion in a previous pregnancy increases the risk of marginal insertion in the subsequent pregnancy (and vice versa; Table 3). This suggests that these placental conditions share etiologic factors, and supports the assumption that velamentous and marginal cord insertions represent a continuum of conditions that occur as a consequence of an altered placental development. The increased risk of recurrence, the increased risk in the female foetus (Table 1) and the associated risk of malformations (Table 6) suggest that the formation of the placenta and cord is a highly adaptive process that can be modified by environmental factors, maternal characteristics and the properties of the conceptus itself.

Previous studies have produced conflicting findings regarding the association between abnormal cord insertion and foetal malformations [3], [9], [10], [14]. The present population-based study revealed an association between marginal insertion and foetal malformation; this association was stronger when the cord insertion was velamentous (Table 6).

The concept of “trophotropism” has been introduced to explain the preferential development of the placental tissue at sites for optimal uterine perfusion, and more or less distant from the original insertion site [8]. Our finding that abnormal cord insertion was associated with an increased risk of other placental conditions such as placental abruption, placenta praevia, pre-eclampsia and intrauterine growth restriction (Tables 4 and 6) suggests shared genetic and environmental mechanisms associated with altered implantation, migration, invasion and transformation of the spiral arterioles. Such skewed placentation may have a negative impact on the health of both the foetus and mother. The shared pathophysiologic mechanisms of these conditions are further supported by biochemical studies. Both velamentous cord insertion and pre-eclampsia have been shown to exhibit a pattern of increased serum human chorionic gonadotrophin and reduced α-foetoprotein in mid trimester analyses [15], [16].

Our finding of increased risk of recurrence of these placental conditions in a subsequent pregnancy is consistent with another placental study showing that prior placental abruption increases the risk of later placenta praevia [17]. We did not find that anomalous cord insertion in one pregnancy was associated with an increased risk of placenta praevia or abruption in a subsequent pregnancy, which may be due to the low incidence of these conditions.

Women with a prior caesarean had an increased risk of anomalous cord insertion (Table 1), which is in contrast to previous findings [3]. Multiparous women have been shown to have an increased risk of placental abruption and placenta praevia [18]. However, in the present study the first childbirth seemed to have an increased risk of anomalous cord insertion (Table 1), which is in agreement with a previous study [2].

Smokers had a slightly increased risk of velamentous cord insertion, whereas the group that did not answer the smoking question had a reduced risk compared with non-smokers, even after adjustment for maternal age and parity (Table 1). We found that birthweight was lower in the non-responder group than in the non-smoking group. This finding does not support an overrepresentation of non-smokers among the non-responders, instead suggesting effects of other confounding factors.

The present study confirms the previous finding of increased risk of preterm birth in pregnancies with anomalous cord insertion, whereas the risk of pre-eclampsia was only increased in the term group. The increased risk of emergency caesarean delivery for pregnancies with velamentous or marginal insertion of the cord (Table 5)–in view of the unchanged risk of operative vaginal delivery–suggests that abnormal cord insertion is likely to lead to intervention before the second stage of labour.

The birthweight/placental-weight ratio varies with placental proportions and is linked to functional variations [19]. Different patterns of placental growth–in terms of the chorionic disk area and thickness–lead to different birthweights for a given placental weight, indicating that foetal growth is dependent on placental mass, organization and nutrient-transfer capacity. The vessel density is lower in placentas with abnormal cord insertion than in those with normal cord insertion [7], and the foetal stem vessels may be longer in the former, which would increase vascular resistance and hamper nutrient transfer. The relationship between the placental weight and the foetal weight changes through gestation [19], [20]. We therefore used percentiles to assess placental weights and birthweights and their interrelationship. The risk of having a small placenta (i.e. <10^th^ percentile) was increased in anomalous cord insertion, while there was very little risk of a low birthweight/placental-weight ratio (Table 5).

The placental pathology in velamentous and marginal cord insertions may partly explain the increased perinatal risk associated with these conditions. We did not assess the diagnoses of perinatal morbidity in further detail, but one obvious explanation for increased perinatal risk in pregnancies with velamentous cord insertion is the acute cessation of umbilical blood flow due to pressure on vessels not protected by Wharton’s jelly, or disruption of vessels causing foetal bleeding. Animal experiments suggest that umbilical compression reduces cardiac output and increases the risk of pulmonary complications after birth [21]–[23]. The placental insufficiency accompanying abnormal cord insertion may increase the susceptibility to insults caused by the compression of velamentous vessels. The fraction of the combined foetal cardiac output directed to the placenta is reduced to 20% during the last weeks of pregnancy, while 80% is recirculated within the foetal body [24], thus lowering the physiologic reserves during this period. This recirculation is augmented in pregnancies with growth restriction, further reducing the foetal reserves. Such factors could explain why we found an increased risk at term for caesarean delivery, low Apgar score, and perinatal death associated with velamentous cord insertion.

It has been shown that birthweight and cord and placental characteristics are gender dependent [7], [13], [17], [19], [25], [26]. In contrast to another study [3], we found an increased risk of anomalous cord insertion in female foetuses.

The observed increased risk of anomalous cord insertion among twins is well documented [11], [12], [27]. Monochorionic placentas have an increased risk of velamentous cord insertion [28], [29], and non-central cord insertion is more frequent in the smaller foetus in twins exhibiting discordant growth [11]. The findings of the present study support this finding, but do not permit further elaboration since chorionicity was not recorded in the registry. However, it is known that an increased risk of poor perinatal outcome is linked to both twins in monochorionic pregnancies with velamentous cord insertion [30], and that cord insertion site influence the birthweight in all types of twins [11], [31]–[33].

Another study based on the Medical Birth Registry of Norway showed that multivitamin and folate supplementation was associated with reduced risk of placental abruption [34]. In the present study we found conflicting results, with only a borderline significant effect of supplementation (i.e. increased risk of velamentous but reduced risk of marginal cord insertion; Table 2).

The strength of our study is that it was population based, and that the size of the cohort was sufficient to investigate rare expositions and outcomes such as perinatal death. The data set allowed us to examine the rate of recurrence and to adjust for possible confounding factors. However, the use of registry data carries risks of misclassification and selection bias. One such source may be the special attention given to the placenta and cord insertion when the outcome is not normal. The development of an abnormal cord insertion is probably influenced by many factors, and in our study it was only possible to control for factors that are entered in the registry. However, several variables in the registry have been validated, suggesting satisfactory internal validity [35]–[37]. The nature of our dataset cannot answer the question of chronology of all events, for instance is bleeding in pregnancy caused by anomalous cord insertion, or does a pathological placentation underlie both anomalous cord insertion and bleeding? Since bleeding irrespective of gestational age is associated with anomalous cord insertion we find the latter hypothesis the most plausible.

Prenatal identification of anomalous cord insertion is feasible [38], but whether this would reduce the risk of adverse outcomes needs further investigation.

## Conclusion

The present population-based study found prevalence rates of 1.5% and 6.3% for velamentous and marginal cord insertions, respectively, in singleton pregnancies, and higher rates in multiple pregnancies. The results suggest that the two conditions are closely related and have common risk factors, but in a graded fashion, with the velamentous insertion being the more-severe condition. Abnormal insertion of the cord triples the risk of perinatal death at term, which may justify an increased focus during pregnancy to identify this condition in order to better prepare for the care provided at term.

## Supporting Information

Table S1 **Descriptive statistics of 11263 twin pregnancies.**
(DOCX)

List S1 **List of diagnoses in the ICD10 system classified as serious malformations in the Medical Birth Registry of Norway.**
(DOCX)
