# Supplementary material for: Prevalence, Risk Factors and Outcomes of Velamentous and Marginal Cord Insertions: A Population-Based Study of 634,741 Pregnancies
Source: PLoS One. 2013 Jul 30;8(7):e70380. doi: 10.1371/journal.pone.0070380 (PMC3728211; doi:10.1371/journal.pone.0070380)
Supplement: Table S1 — Descriptive statistics of 11263 twin pregnancies. (DOCX) [file pone.0070380.s001.docx]

**Table S1:** Descriptive statistics of 11263 twin pregnancies

| **Maternal and pregnancy characteristics** |  | **Frequency (n)** | **%** |
| --- | --- | --- | --- |
| **Gestational age at birth (weeks)** | 17-21 | 113 | 1.0 |
|  | 22-26 | 232 | 2.1 |
|  | 27-31 | 735 | 6.5 |
|  | 32-36 | 4301 | 38.2 |
|  | 37-42 | 5875 | 52.2 |
|  | 43- | 7 | 0.1 |
| **Parity** | 0 | 5016 | 44.5 |
|  | 1 | 3866 | 34.3 |
|  | 2 | 1639 | 14.6 |
|  | 3 | 524 | 4.7 |
|  | >3 | 218 | 1.9 |
| **Maternal age (years)** | <20 | 101 | 0.9 |
|  | 20-24 | 961 | 8.5 |
|  | 25-29 | 3075 | 27.3 |
|  | 30-34 | 4508 | 40.0 |
|  | 35-39 | 2307 | 20.5 |
|  | 40+ | 310 | 2.8 |
|  | Unknown | 1 | 0 |
| **Smoking at the start of pregnancy** | No | 7591 | 67.4 |
|  | Yes | 1690 | 15.0 |
|  | NA | 1982 | 17.6 |
| **Assisted reproductive technology** | No | 8880 | 78.8 |
|  | Yes | 2383 | 21.2 |
