# Supplementary material for: Prevalence, Risk Factors and Outcomes of Velamentous and Marginal Cord Insertions: A Population-Based Study of 634,741 Pregnancies
Source: PLoS One. 2013 Jul 30;8(7):e70380. doi: 10.1371/journal.pone.0070380 (PMC3728211; doi:10.1371/journal.pone.0070380)
Supplement: List S1 — List of diagnoses in the ICD10 system classified as serious malformations in the Medical Birth Registry of Norway. (DOCX) [file pone.0070380.s002.docx]

ICD10 diagnoses: Q000-Q139; Q048; Q054; Q059; Q078-Q079; Q111-Q112; Q120; Q130-Q131; Q15; Q172; Q180 Q182; Q188; Q200-Q289; Q30; Q318; Q330; Q333; Q350-Q379; Q39; Q400; Q408; Q410-Q459; Q500-Q529; Q54; Q557-Q558; Q56; Q601; Q606; Q62; Q634; Q640-Q641; Q643; Q648; Q660; Q680; Q71; Q743; Q75; Q77; Q780; Q790; Q792-Q793; Q813; Q850-Q851; Q89; Q9; P835
